# Use of Community-Based Participatory Research in Mental Health Studies With Older Adults: A Systematic Review

**DOI:** 10.1093/geroni/igab046.1941

**Published:** 2021-12-17

**Authors:** Jessie Ho-Yin Yau, Walker Siu Hong Au, Tianyin Liu, Anna Y Zhang, Gloria H Y Wong, Terry Y S Lum

**Affiliations:** 1 The University of Hong Kong, Hong Kong, Hong Kong; 2 The University of Hong Kong, The University of Hong Kong, Hong Kong

## Abstract

Community-based participatory research (CBPR), a bottom-up approach that community stakeholders and academics are involved equitably, is an effective approach for enhancing relevance and value in public health research and has gained popularity in recent decades. However, little is known about how CBPR can be used in mental health studies with older adults. This systematic review examined the current state of knowledge about how CBPR approach has been adopted in mental health research among older adults in different societies. According to the PRISMA guidelines, we searched five major databases and screened the literature using these criteria: 1) journal articles reporting use of CBPR in mental health research among older adults, 2) articles published in English language, 3) studies conducted in any settings with any mental health research. Initial search found 3,227 articles and preliminary screening identified 23 eligible articles. We found that around 90% of studies were conducted in the West. Most studies adopted CBPR to develop community-based mental health interventions or to revise current interventions or models while addressing the cultural needs of their studied population. Few studies adopted CBPR to evaluate existing mental health workshops or programmes. The extent of involvement of older adults in the CBPR approach varied across studies, from questionnaire design to programme evaluation. Our review uncovered ways of CBPR implementation across different societies and elements of successful implementation in CBPR practices in mental health research among older adults.

